# Three-Dimensional Quantification of Collagen Microstructure During Tensile Mechanical Loading of Skin

**DOI:** 10.3389/fbioe.2021.642866

**Published:** 2021-03-03

**Authors:** Alan E. Woessner, Jake D. Jones, Nathan J. Witt, Edward A. Sander, Kyle P. Quinn

**Affiliations:** ^1^Department of Biomedical Engineering, University of Arkansas, Fayetteville, AR, United States; ^2^Department of Biomedical Engineering, University of Iowa, Iowa City, IA, United States

**Keywords:** skin, collagen, multiscale, microstructure, kinematics, second harmonic generation, structure-function relationship

## Abstract

Skin is a heterogeneous tissue that can undergo substantial structural and functional changes with age, disease, or following injury. Understanding how these changes impact the mechanical properties of skin requires three-dimensional (3D) quantification of the tissue microstructure and its kinematics. The goal of this study was to quantify these structure-function relationships *via* second harmonic generation (SHG) microscopy of mouse skin under tensile mechanical loading. Tissue deformation at the macro- and micro-scale was quantified, and a substantial decrease in tissue volume and a large Poisson’s ratio was detected with stretch, indicating the skin differs substantially from the hyperelastic material models historically used to explain its behavior. Additionally, the relative amount of measured strain did not significantly change between length scales, suggesting that the collagen fiber network is uniformly distributing applied strains. Analysis of undeformed collagen fiber organization and volume fraction revealed a length scale dependency for both metrics. 3D analysis of SHG volumes also showed that collagen fiber alignment increased in the direction of stretch, but fiber volume fraction did not change. Interestingly, 3D fiber kinematics was found to have a non-affine relationship with tissue deformation, and an affine transformation of the micro-scale fiber network overestimates the amount of fiber realignment. This result, along with the other outcomes, highlights the importance of accurate, scale-matched 3D experimental measurements when developing multi-scale models of skin mechanical function.

## Introduction

Skin is a complex organ that provides a protective external barrier for the underlying musculoskeletal tissues of the human body during daily living. Understanding the biomechanics of skin is important for preventing injury, planning clinical procedures, engineering wearable technologies, improving skin care products, and understanding aging (Aubert et al., 1985; Sen et al., 2009; Hussain et al., 2013; Alberto et al., 2018; Blair et al., 2020). The mechanical response of skin has been extensively studied in the past. Through studies involving mechanical testing and histology, collagen fibers within the dermis have been identified as the primary load bearing structure in skin (Fackler et al., 1981; Blair et al., 2020). Additionally, the relative organization of collagen fibers plays a key role its anisotropic mechanical behavior (Billiar and Sacks, 1997; Lake et al., 2012). To better understand the relationship between collagen fiber organization and the mechanical function of soft tissues, substantial advancements involving computational modeling that incorporate the collagen microstructure have been made over the last 20 years (Fung, 1967; Sander et al., 2009; Fang and Lake, 2016; Limbert, 2017). The development of these computational models has outpaced experimental measurements of the collagen microstructure under mechanical loading, and many assumptions regarding the fiber and tissue kinematics at the microscale remain untested, which limits our understanding of tissue structure-function relationships (Fang and Lake, 2016).

Historically, histological staining of tissue has been the gold standard for evaluating skin microstructure and its relationship to biomechanics (Ridge and Wright, 1966; Lanir, 1979; Jor et al., 2013; Pensalfini et al., 2018). However, this assessment of microstructure is inherently destructive due to the sample preparation process and cannot be used to track tissue or fiber kinematics during mechanical loading. Digital image correlation (DIC) has previously been used in various studies of skin to observe interesting macro-scale tissue kinematics, such as a large Poisson’s ratio and a decrease in volume during uniaxial mechanical stretching, but these techniques cannot be used to quantify fiber kinematics (Hepworth et al., 2001; Pissarenko et al., 2019; Wahlsten et al., 2019; Pissarenko and Meyers, 2020). To understand the relationship between collagen fiber kinematics and the tissue’s mechanical response, imaging methods such as quantitative polarized light imaging (QPLI) and small angle light scattering (SALS) have been integrated with mechanical testing (Sacks et al., 1997; Tower et al., 2002; Quinn and Winkelstein, 2008; Sander et al., 2009; Woessner et al., 2019). Instead of visualizing individual fibers, however, these methods infer the average orientation and degree of alignment of thousands to millions of fibrils with macro-scale resolutions on the order of millimeters. These approaches provide a dynamic two-dimensional (2D) summary of fiber kinematics and organization during mechanical loading. However, collagen fibers are organized in a three-dimensional (3D) matrix that can vary as a function of depth, and skin and other soft tissues have differences in organization among all three-dimensions (Roeder et al., 2002; Limbert, 2017).

To better understand the reorganization of collagen fibers during mechanical loading, 2D distributions of the micro-scale collagen fiber reorganization in human tendon (Lake et al., 2012), bovine pericardia (Sacks, 2003), and porcine mitral valve leaflets (Lee et al., 2015) have been found to share an affine relationship with tissue deformation. When similar experiments were performed on porcine (Hepworth et al., 2001) and mouse (Bancelin et al., 2015) skin, the 2D micro-scale distribution of collagen fiber reorientation did not match the distributions one would predict if the fiber kinematics were affine with the macro-scale tissue deformation. Furthermore, previous 3D measurements of multi-scale strain in collagen gels revealed that the magnitude of micro-scale strain in the direction of stretch was lower than the amount of applied strain at the macro-scale, indicating a need to quantify both 3D fiber kinematics and organization at the micro-scale in skin (Roeder et al., 2009). In summary, a more detailed 3D description of the multi-scale mechanical behavior of skin, particularly with respect to its microstructure, is still needed to inform, validate, and refine appropriate computational and constitutive models.

Depth-resolved optical microscopy approaches offer both 3D imaging capabilities and sub-micrometer resolution capable of non-destructive visualization of individual fibers within a tissue. One such imaging approach, reflectance confocal microscopy, has been used to characterize multiscale deformation and fiber kinematics in 3D collagen gels during mechanical stretch, showing that the relative magnitude of micro-scale strain can be quite different from the amount of strain experienced at the bulk tissue scale (Roeder et al., 2002, 2004, 2009; Voytik-Harbin et al., 2003). However, soft biological tissues are more complex than collagen gels, with more heterogeneity in matrix structure and composition and a greater density of extracellular matrix (ECM) proteins. Due to the high density of matrix components in native tissues, reflectance confocal microscopy has limited depth penetration and lacks the ability to discriminate compositional elements in skin. Multiphoton microscopy (MPM) is a non-linear optical microscopy technique that has greater depth penetration than confocal microscopy due to the use of near-infrared light (Yasui et al., 2013). Through MPM, collagen fibers can be visualized at the micro-scale with high specificity based on a strong second harmonic generation (SHG) signal, and two-photon excited fluorescence (TPEF) from endogenous cellular fluorophores and other ECM proteins can provide additional context during MPM imaging (Quinn et al., 2016). Recently, SHG has been utilized to measure 2D micro-scale fiber kinematics and organization of mouse skin during mechanical testing, but these experiments were performed only on the dermal layer of the skin, and have yet to characterize fiber kinematics and tissue deformation in 3D (Bancelin et al., 2015; Lynch et al., 2017a,b).

The goal of this study was to quantify 3D fiber organization and kinematics at the micro-scale, as well as characterize tissue kinematics across multiple length-scales during tensile loading of skin. To this end, a uniaxial mechanical testing device was integrated with a multiphoton microscope to quantitatively assess macro- and micro-scale kinematics simultaneously during mechanical stretch. 3D analysis of micro-scale fiber kinematics and organization was performed using established analysis techniques. Macro-scale kinematics were calculated through analysis of fiduciary markers and tissue kinematics were compared across length-scales. This multi-scale analysis of skin provides new insights into how the 3D microstructure responds to tensile loading, which is critical for developing and refining computational models that relate tissue structure and mechanical function.

## Materials and Methods

### Sample Preparation

Full thickness ventral skin from male C57BL/6J mice (4 months old, *n* = 7) was collected, snap-frozen, and stored at −80°C until mechanical testing. Prior to testing, tissue samples were thawed and prepared by cleaning the skin with an alcohol wipe and carefully resecting the hypodermis. Traditional dog-bone shapes were cut from the skin in the medial-lateral direction using a precision-machined steel stamp (26 mm × 8 mm total shape size, 6 mm × 2 mm gauge region) (Sander et al., 2014), and four fiduciary markers were added to the tissue gauge region using a fine-tipped ink pen to enable macro-scale analysis of tissue kinematics.

### Imaging System

**FIGURE 1:**
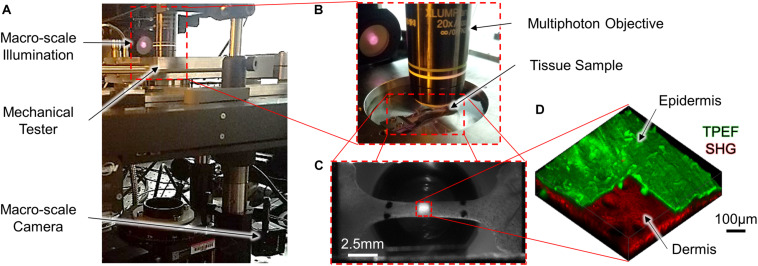
A mechanical tensile testing device integrated with a multi-scale imaging system **(A,B)** was used to collect macro-scale 2D images [**(C)**; scale bar represents 2.5 mm] and micro-scale 3D MPM image volumes [**(D)**; scale bar represents 100 μm]. To highlight the difference in scale between the macro- and micro-scale ROIs, the red dashed square in the center region of panel **(C)** represents the relative size of the ROI collected in panel **(D)**. At the micro-scale, both SHG and TPEF image volumes were collected, and the pseudo-colored TPEF volume shown in panel **(D)** is cutout to highlight the layers of the skin.

To acquire multi-scale image data during mechanical testing, a uniaxial mechanical tester (ADMET BioTense; ADMET; Norwood, MA, United States) integrated into a multiphoton microscope and macro-scale imaging system was used (Figures 1A,B). Macro-scale imaging was performed by illuminating the tissue with two near-infrared mounted 780 nm LEDs (M780L3; ThorLabs; Newton, NJ, United States) and the transmitted light was collected with a fixed focal length lens (MVL50M23; ThorLabs; Newton, NJ, United States) and imaged at a rate of 0.2 Hz (2,464 × 2,056 pixels, 0.02 mm/pixel) with a camera (Lt525R; Lumenera Corporation; Ottawa, ON, United States) controlled through custom-written MATLAB code (MathWorks; Natick, MA, United States) (Figure 1C). For micro-scale imaging, an upright multiphoton microscope (Investigator series; Bruker; Billerica, MA, United States) equipped with a tunable ultrafast pulsed laser (InsightX3; Spectra Physics; Mountain View, CA, United States) and 20x, 1.0 NA water-dipping objective (XLUMPLFLN20XW; Olympus; Tokyo, Japan) was used to collect SHG (800 nm excitation, 400 nm emission) and TPEF (755 nm excitation, 460 nm and 525 nm emission) image z-stacks in the epi-direction. (512 × 512 × 130 pixels, 1 μm z-steps, 1.144 μm/pixel X-Y resolution) (Figure 1D).

### Data Acquisition

**FIGURE 2:**
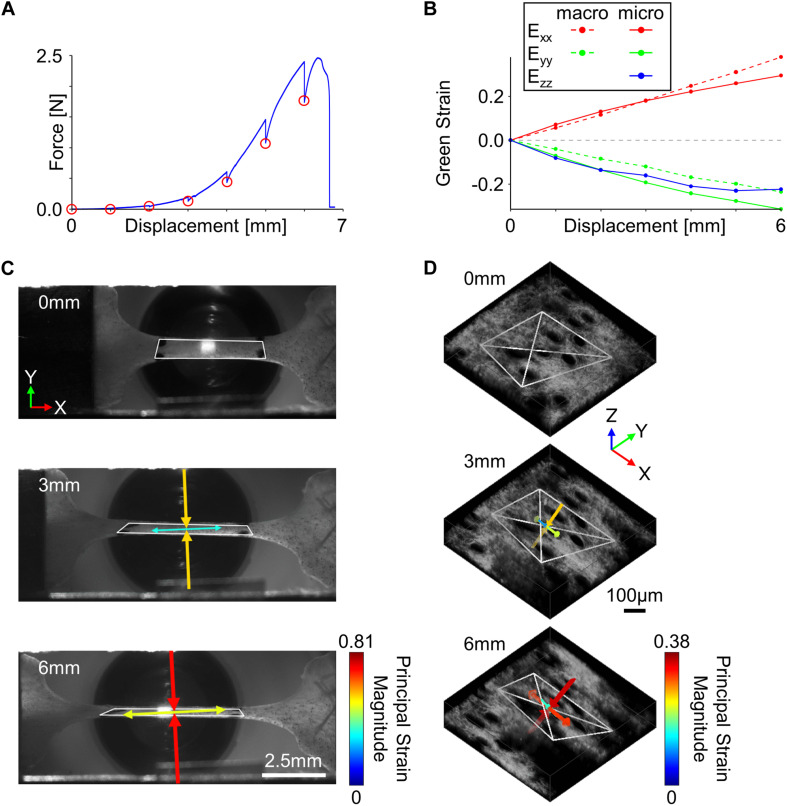
Tissue kinematics were quantified at multiple length scales during incremental stretching of skin in the x-direction. **(A)** To enable the collection of high-resolution micro-scale SHG image volumes, the skin was stretched in 1 mm increments (red circles) until mechanical failure. **(B)** Tissue kinematics were restricted to 2D at the macro-scale but were quantified in 3D at the micro-scale. **(C)** Macro-scale tissue deformation measured from fiduciary markers showed a substantial compressive principal strain orthogonal to the direction of stretch (scale bar represents 2.5 mm). **(D)** 3D micro-scale tissue kinematics measured from SHG features indicated compressive principal strains were occurring in both directions orthogonal to the loading direction (scale bar represents 100 μm). For panels **(C,D)**, white polygons indicate tracked features, and arrows represent the direction and magnitude of principal strains.

Tissue samples loaded in the uniaxial mechanical testing device were submerged in a bath filled with phosphate buffered saline (PBS) to ensure hydration during the duration of the test. Prior to loading, the tissue geometry was optically measured using the macro-scale imaging system and pre-stressed to 10 kPa, which was considered the undeformed configuration for all samples (Quinn and Winkelstein, 2008). Tissue samples were preconditioned to ∼5% strain (20 cycles, 4 mm/min) to ensure a repeatable mechanical response (Giles et al., 2007; Quinn and Winkelstein, 2011), and the allowed to rest for 5 min to allow for hydrostatic recovery. Representative SHG and TPEF image volumes at three discrete locations spanning approximately the length of the gauge region were then collected to assess the undeformed microstructure. During mechanical testing, tissues samples were stretched in 1 mm increments at a quasi-static rate of 0.2 mm/min (Figure 2A). The initially undeformed ROI taken within the center of the gauge region was visually tracked during mechanical stretching *via* SHG imaging (∼1.2 Hz), and at every 1 mm increment, the mechanical stretching was stopped to allow for collection of a high-resolution SHG image z-stack of the ROI (Bancelin et al., 2015).

### Analysis of Deformation

At the macro-scale, fiduciary markers were tracked within the camera images using an automated normalized cross-correlation algorithm, and the deformation gradient (*F*) was computed at every 1 mm increment of displacement using isoparametric mapping (Quinn and Winkelstein, 2009). The deformation gradient was then used to calculate the 2D Green strain tensor (*E*) (Figure 2B),

$$E=0.5\,\left(F^{T}\cdot F-I\right),$$

where *I* is the identity tensor. Principal strain magnitudes and directions were computed as the eigenvalues and eigenvectors of the 2D strain tensor, respectively (Figure 2C; Quinn and Winkelstein, 2008; Oñate, 2009; Nikishkov, 2010). Using the 2D strain tensor measurements, the incremental Poisson’s ratio (ν),

$$\nu =-E_{y\,y}/E_{x\,x},$$

was calculated for each tissue sample during loading. Due to the Poisson’s ratio being a material property, all incremental Poisson’s ratio measurements for a particular sample were averaged together.

To calculate micro-scale tissue kinematics, trackable collagen fiber patterns were first identified using a 3D extension of the Harris corner detection algorithm (Harris and Stephens, 1988). Pixels surrounding each fiber location selected by the corner detection algorithm were stored (21 × 21 × 11 pixels), and at every 1 mm increment of displacement, locations were tracked in 3D. Accurate and precise tracking of 3D features consisted of first computing the position of current features (*x_*t*_, y_*t*_, z_*t*_*) in the next sequential image volume (*x_*t*+1_, y_*t*+1_, z_*t*+1_*) using a 3D normalized cross-correlation algorithm. Pixels surrounding the (*x_*t*+1_, y_*t*+1_, z_*t*+1_*) position were then collected and used to recalculate the feature position in the current image volume (*x_*t*_’, y_*t*_’, z_*t*_’*). Feature displacement was then calculated as the average displacement between the (*x_*t*+1_, y_*t*+1_, z_*t*+1_*) and (*x_*t*_’, y_*t*_’, z_*t*_’*) vectors, and the amount of tracking error was calculated as the Euclidean distance between the (*x_*t*_, y_*t*_, z_*t*_*) and (*x_*t*_’, y_*t*_’, z_*t*_’*) vectors. To filter out features that did not correctly track among all image volumes, only features with a maximum tracking error of <5 pixels for the entire mechanical test were considered for analysis (Quinn and Winkelstein, 2010). Since at least four features are required to calculate micro-scale deformation in 3D, only image volumes that contained at least four correctly tracked features were used for analysis. To get a 3D measurement of micro-scale deformation, 3D tetrahedrons were generated from all possible combinations of correctly tracked features, and their volumes were calculated in the undeformed state. The four feature locations that made up the largest possible tetrahedron based on volume were used to calculate micro-scale tissue kinematics (Figure 2D). Similar to the macro-scale analysis, the deformation gradient at each 1 mm increment was calculated using 3D isoparametric mapping (Nikishkov, 2010), and a 3D Green strain tensor was calculated at each 1 mm increment (Figure 2B). Principal strain magnitudes and directions were computed as the eigenvalues and eigenvectors of the Green strain tensor, respectively (Figure 2D). To quantify how the micro-scale volume changed as a function of stretch, a volume ratio was calculated from the deformation tensor at each 1 mm of displacement,

$$V/V_{0}=\text{det}\,\left(F\right).$$

### Analysis of Fiber Organization and Volume Fraction

**FIGURE 3:**
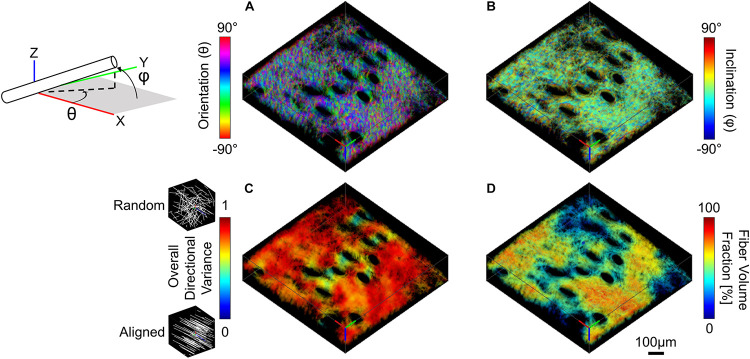
SHG image volumes were used to compute a pixel-wise map of 3D micro-scale orientation **(A)** and inclination **(B)**. The two 3D orientation and inclination maps and collagen-positive mask were then convolved with a range of cubic averaging kernels varying in size to determine how regional measurements of directional variance **(C)** and fiber volume fraction **(D)** change as a function of the length scale over which they are measured (convolution with 40 μm^3^ kernel shown here, scale bar represents 100 μm).

Three-dimensional collagen fiber orientation for SHG image volumes was measured using a weighted vector summation algorithm as previously described (Quinn and Georgakoudi, 2013; Liu et al., 2017). Calculation of pixel-wise 3D fiber orientation was based on a surrounding 7 × 7 × 7 pixel window and resulted in a pixel-wise map of spherical coordinates, θ (X-Y plane orientation) and φ (Z axis inclination) (Figures 3A,B). Fibers within the image voxel were then isolated by first computing an average SHG intensity for the entire image volume, and all pixels with intensity greater than or equal to the average voxel intensity were used to create a collagen-positive volumetric mask. To assess fiber alignment during stretch, directional variance based on the 3D orientation of all fiber-containing pixels within an image volume was calculated. Directional variance is bounded by values of zero and one, which correspond to an aligned and random fiber network organization, respectively (Figure 3C; Liu et al., 2017). Additionally, a fiber volume fraction was calculated as the ratio between the number of collagen-positive pixels and the total number of pixels within an image volume.

To assess whether fiber alignment during stretch was affine with the local tissue deformation, comparisons were made between the measured fiber directional variance at peak strain and the theoretical affine fiber kinematics at peak strain. The measured 3D deformation gradient tensor was applied to the undeformed image volume using built-in MATLAB functions (*affine3D* and *imwarp*) to produce theoretical image volumes at peak strain under an affine assumption (Fan and Sacks, 2014; Jayyosi et al., 2017). As was the case with the actual fiber kinematics, maps of 3D fiber orientation were computed from the theoretical affine image volumes. To compare fiber organization for both measured and theoretical deformed image volumes, 3D directional variance was calculated for all fiber-containing pixels within the 3D tetrahedron formed from the tracked features. Additionally, 2D directional variance was calculated for both fiber orientation (θ) and inclination (φ) (Figures 3A,B) to determine whether the fiber kinematic response was affine with the local deformation in all planes.

Collagen fiber directional variance has previously been found to vary as a function of the length scale over which it is measured (Quinn et al., 2015). For this study, fiber directional variance and volume fraction at the micro-scale was measured at multiple length scales within the image volume by convolving the pixel-wise fiber orientation and inclination maps, as well as the fiber-containing mask with cubic averaging kernels of varying sizes (4^3^, 10^3^, 20^3^, 40^3^, 70^3^, and 100^3^ μm^3^). This convolution process results in a regional summary of directional variance and fiber volume fraction, which was then averaged and compared among length-scales (Figures 3C,D; Quinn et al., 2015; Woessner et al., 2019).

### Statistical Analysis

At both the macro- and micro-scale, the tissue kinematics were compared to the expected outcomes of an incompressible material (*ν* = 0.5 for Poisson’s ratio, V/V_0_ = 1 for volume ratio) through a one sample *t*-test. Comparisons of strain across the two length scales, as well as between undeformed and deformed (at peak strain) micro-scale organization and fiber volume fraction were performed using paired student’s *t*-tests. Additionally, comparisons of directional variance between theoretically and mechanically deformed image volumes were performed using paired student’s *t*-tests. To assess the length scale dependencies at the micro-scale, comparisons were performed with one-way ANOVAs and *post hoc* Tukey’s HSD tests to determine significant differences. Significant differences were defined as a *p*-value < 0.05, and all statistical analysis was done using JMP Pro 13 (SAS Institute; Cary, NC, United States).

## Results

### Tissue Deformation

**FIGURE 4:**
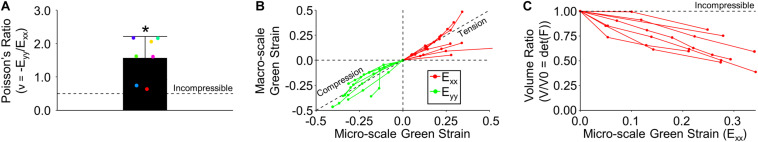
Tissue kinematics measured at multiple length scales were found to experience relatively similar magnitudes of strain and exhibit compressible material behaviors. **(A)** At the macro-scale, tissue exhibited a very large Poisson’s ratio that was significantly different than expected from an incompressible material. **(B)** Macro- and micro-scale Green strain for all samples indicates similar tissue deformation across length-scales. **(C)** Tissue underwent a significant decrease in micro-scale volume during stretch.

At the macro-scale, skin under uniaxial tension (in the x-direction) exhibited a significant inward orthogonal contraction (in the y-direction) (Figure 2C), producing an average Poisson’s ratio (1.57 ± 0.65) that was significantly greater than that of an incompressible material (*p* = 0.004) (Figure 4A). When compared across length-scales, micro- and macro-scale strain measurements did not significantly differ in either the E_*xx*_ or E_*yy*_ directions (*p* = 0.26 and *p* = 0.44, respectively) (Figure 4B). Interestingly, the 3D deformation gradient measured *via* MPM image feature tracking revealed a substantial decrease in tissue volume ratio (V/V_0_) with increasing strain that was significantly different from the expected response of an incompressible material (*p* = 0.0003) or other rubber-like material models historically used to model soft biological tissue (Figure 4C; Fung, 1967; Crichton et al., 2013).

### Micro-Scale Fiber Organization and Volume Fraction

**FIGURE 5:**
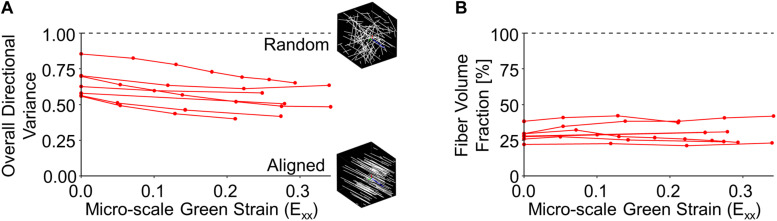
Collagen fiber directional variance decreased during stretch **(A)**, indicating increased alignment, but fiber volume fraction did not significantly change **(B)**.

The volume fraction and alignment of collagen fibers can provide some context for the large changes in tissue volume observed during loading. At the micro-scale, initial collagen fiber directional variance for the entire image volume (0.654 ± 0.107) was significantly higher than at peak strain (0.524 ± 0.100; *p* = 0.002), indicating increased fiber alignment with stretch (Figure 5A). Despite significant reductions in the macro-scale tissue volume, no significant difference between the undeformed fiber volume fraction (28.6 ± 4.95%) and that at peak strain (30.1 ± 7.35%; *p* = 0.524) (Figure 5B) was found.

**FIGURE 6:**
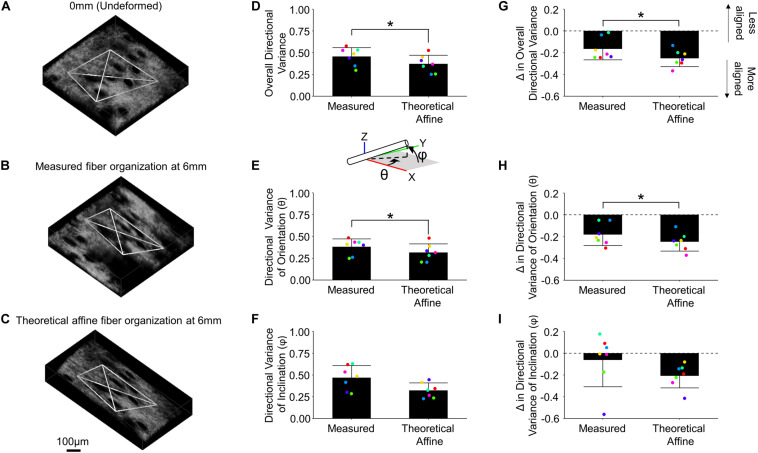
Collagen fiber kinematics are not affine with the local skin deformation. Micro-scale image volumes were compared among the undeformed configuration **(A)**, actual tissue response at peak strain **(B)** and the theoretical affine transformation **(C)** based on the measured deformation gradient. The white tetrahedron in panels **(A–C)** indicates the position of 3D image features that were tracked during deformation (scale bar represents 100 μm). The computed 3D directional variance **(D)** as well as the measured directional variance for individual 3D orientation and inclination components [θ and φ, panels **(E,F)**, respectively] were found to be higher than the theoretical affine transformation. Similarly, the change in overall directional variance **(G)** and the 3D orientation and inclination components **(H,I)** indicate the measured fiber re-alignment in the direction of loading is less than the theoretical affine response. Points are colored to indicate paired comparisons among groups. *represents *p* < 0.05.

In order to determine whether fiber reorganization was affine with stretch or not, the experimentally measured fiber kinematics in the mechanically deformed images were compared to the kinematics resulting from an affine transformation of the same images (Figures 6A–C). The measured 3D directional variance within the tetrahedron in mechanically deformed image volumes (0.457 ± 0.101) was significantly higher (*p* = 0.012) than the variance under a theoretical affine transformation (0.372 ± 0.100) (Figure 6D). These differences were conserved along multiple anatomical planes. A higher average directional variance was measured for orientations in the x-y plane (θ) during deformation (0.380 ± 0.091; *p* = 0.015) compared to the theoretical affine transformation (0.314 ± 0.099) (Figure 6E). A higher average directional variance was also measured (0.467 ± 0.141) for fiber inclination out of the x-y plane (φ) compared to the theoretical affine transformation (0.322 ± 0.086), but these changes did not meet the criteria for statistical significance (*p* = 0.056) (Figure 6F). When these results are viewed as a change in directional variance relative to the undeformed configuration (Figures 6G–I), it becomes apparent that the actual fiber realignment in the direction of loading is less than what was calculated for an affine transformation.

**FIGURE 7:**
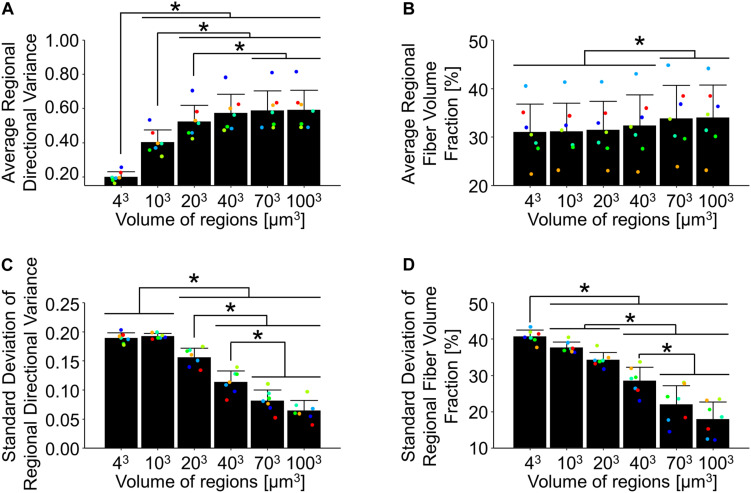
In the undeformed configuration, regional averages of directional variance **(A)** and fiber volume fraction **(B)** increased as a function of the length scale over which they were measured. Alternatively, the average regional standard deviations of directional variance **(C)** and fiber volume fraction **(D)** decreased at larger length-scales, indicating the importance of length-scale when implementing measurements into computational models. Points are colored to indicate paired comparisons among groups. *represents *p* < 0.05.

Dermal collagen is often described as having a basket-weave like organization, which has previously been found to vary in histological sections as a function of the length scale over which it is measured (Fisher et al., 2008). To quantify this variability in 3D SHG image volumes, the average and standard deviations of regional fiber volume fraction and directional variance were computed over different scales within each undeformed SHG image volume. When the average regional directional variance was compared across length scales, it was found to significantly increase with increasing length scale (*p* ≤ 0.01) until it plateaued around a scale of 40 μm^3^ (Figures 3C, 7A). Interestingly, the standard deviation of regional directional variance was not significantly different between volumes of 4^3^ and 10^3^ μm^3^ (*p* = 0.99), but it did decrease significantly as the length scale increased from this range (*p* ≤ 0.001) (Figure 7C). The average regional fiber volume fraction remained constant from 4^3^ to 40^3^ μm^3^ (*p* ≥ 0.09), followed by a slight increase at larger length scales (*p* < 0.001) (Figures 3D, 7B). As length scale increased, the standard deviation of regional fiber volume fraction decreased (*p* ≤ 0.01) (Figure 7D).

## Discussion

In this study, 2D macro-scale and 3D micro-scale tissue kinematics were quantified simultaneously during mechanical stretch. Historically in biomechanics, it has often been assumed that soft tissues are nearly incompressible (Lees et al., 1991). In this study, however, deformation at the macro-scale revealed a very large Poisson’s ratio for mouse skin. Recently others have reported large Poisson’s ratios both computationally (Reese et al., 2010; Ban et al., 2019) and experimentally in tendon (Lynch et al., 2003; Lake et al., 2010; Reese and Weiss, 2013), cartilage (Elliott et al., 2002), and skin (Pissarenko et al., 2019; Wahlsten et al., 2019). These observations may be related to a contractile force orthogonal to the direction of stretch caused by collagen fiber realignment (Figure 5A; Ban et al., 2019). The large Poisson’s ratio phenomenon has been identified to be mechanically anisotropic (Elliott et al., 2002) and location dependent (Pissarenko et al., 2019). Although Poisson’s ratio can be estimated during uniaxial stretching, the material is unconstrained in the axis orthogonal to the direction of stretch, and biaxial mechanical stretching of skin is required to fully characterize the force-coupling that exists between multiple axes during stretch. Interestingly, an examination of the tissue’s micro-scale kinematics revealed a substantial decrease in volume (Figure 4C) and significant compression along both directions orthogonal to the direction of stretch. Although previous studies have described a decrease in the micro-scale volume of collagen gels during uniaxial stretch (Roeder et al., 2004), and macro-scale volume decreases in murine and human skin (Wahlsten et al., 2019), a decrease in the micro-scale volume of skin has not been explicitly measured before. Volume loss during deformation is thought to be due to interstitial fluid flowing out of the dermal ECM (Roth and Mow, 1980; Wahlsten et al., 2019), but more research is needed to verify this hypothesis and determine if other mechanisms are involved.

A significant decrease in micro-scale volume was measured during uniaxial stretch in this study, but the fraction of voxels that contain fibers did not change significantly during stretch (Figure 5B). This lack of a change in volume fraction indicates that the collagenous regions of the dermis are decreasing in volume in a similar amount as the non-collagenous regions of the skin during stretch. Given that the mechanical properties of various skin components are different, this result is surprising. However, it is important to consider the resolution of these volume fraction measurements. MPM imaging of collagen fibers *via* SHG is a diffraction-limited microscopy technique that is also limited by the digital resolution of individual voxels during image acquisition (Campagnola and Dong, 2011). It remains quite possible that the actual density of collagen fibers within the SHG-positive voxels may be changing even if the overall volume fraction of SHG-positive voxels does not change. Previous experiments have determined SHG intensity is sensitive to changes in sub-voxel fiber density due to the relationship between measured intensity and fiber diameter (Bancelin et al., 2014). However, we cannot definitively assess fiber density within the SHG-positive voxels based on their SHG intensity in intact skin and other thick samples for a number of reasons. Photon scattering complicates the relationship between SHG intensity and fiber diameter/density (Campagnola and Dong, 2011). Other experiments have also determined that the organization of fibrils within the fiber directly affects the measured SHG intensity due to various phase-matching conditions (Campagnola et al., 2002). Therefore, additional future work is needed to provide a complete assessment of how the density of collagen fibers may change at various scales in intact skin during mechanical stretching.

Previous work that focused on micro-scale deformation of mouse skin under uniaxial stretch was based on the relative position of hair follicles in 2D within SHG image volumes. Additionally, the skin used in those studies (Bancelin et al., 2015; Lynch et al., 2017a,b) was excised from the back of the mouse, stripped of both the hair and epidermis, and was mechanically stretched as a strip of tissue rather than as the traditional dog-bone shape used in our current study. Although micro-scale volume changes were not computed in previous studies by Lynch et al., they measured a much smaller contraction in the transverse direction compared to our current study (Figure 2D), which could be due to differences in excisional location, tissue orientation relative to loading direction, and tissue geometry. Additionally, by utilizing features within the collagen network rather than using hair follicles as reference points for computing the micro-scale deformation, our strain measurements are more reflective of the kinematics and deformation of the collagen microstructure. Multi-scale 3D strain measurements during uniaxial tensile loading of soft tissue are relatively rare, but previous work involving multi-scale strain measurements of collagen gels showed that the amount of micro-scale strain about the axis of stretch was less than at the macro-scale (Roeder et al., 2004). In contrast, no significant differences were found between macro- and micro-scale strain measurements in this study (Figure 4B). However, skin is more complex and denser than typical collagen gels, and multi-scale transmission of strain may be aided by the more complex and dense collagen organization in the dermis or interactions with other layers of the skin (Blair et al., 2020).

To provide context for the changes in tissue strain at the micro-scale, the 3D fiber alignment was quantified during mechanical stretching. During stretch, 3D directional variance measurements in this study showed a significant increase in fiber alignment (Figure 5A), similar to previous findings from 2D fiber organization measurements within skin (Lynch et al., 2017a; Ducourthial et al., 2019). Most computational models of soft tissue kinematics assume that the micro-scale fiber kinematics are primarily driven by tissue-fiber interactions, resulting in an affine relationship between tissue deformation and fiber kinematics (Billiar and Sacks, 1997; Sacks, 2003; Lake et al., 2012; Fan and Sacks, 2014; Lee et al., 2015; Quinn et al., 2016). In skin however, previous findings based on 2D fiber orientation measurements indicated that this relationship is non-affine, and that the affine assumption results in an overestimate of fiber re-organization (Hepworth et al., 2001; Jayyosi et al., 2017). Results from this study (Figure 6D) agree with those findings, showing that the 3D fiber kinematics shares a non-affine relationship with tissue deformation and that the affine assumption overestimates the fiber alignment in the direction of stretch. Our results uniquely show this non-affine fiber behavior in 3D and demonstrates it occurs both in and out of the x-y plane of the skin (Figures 6E,F). The non-affine relationship between tissue deformation and fiber kinematics may be due to fiber-fiber interactions or interactions between the multiple layers of skin or its appendages. To elucidate the key factors driving this non-affine fiber response and understand other complex tissue behaviors, computational modeling will be critical in the future. Modeling efforts have greatly increased in complexity from neo-Hookean strain energy functions proposed decades ago (Fung, 1967) to more recent Fung-type hyperelastic models (Meador et al., 2020) and modern multi-scale simulations involving fiber networks (Sander et al., 2009; Fang and Lake, 2016; Ban et al., 2019). Future work will focus on integrating detailed 3D experimental results with multi-scale image-based computational models of skin mechanical behavior.

In summary, our 3D micro-scale measurements of tissue deformation under uniaxial tension revealed a significant decrease in tissue volume that is contradictory to traditional neo-Hookean or Mooney–Rivlin models previously used in skin applications (Fung, 1967; Weiss et al., 1996). Our 3D measurements of the orientation of individual fibers in this study suggest that fiber kinematics are not affine to the local deformation (Figure 6D). This indicates that models allowing for non-affine kinematics, such as those involving fiber network simulations may be necessary to accurately model the mechanical behavior of skin. We found that summary statistics of collagen fiber alignment and volume fraction are also very dependent on the length scale over which the data are summarized (Figure 7). Due to this length scale dependence, experimental results used to build and test image-based fiber network models need to have appropriate matching length scales (Sander et al., 2009). When interpreting these experimental findings and integrating them with computational modeling, several limitations should be considered. This study focused on the kinematics of collagen fibers, but characterization of other ECM constituents may be important in understanding mechanical phenomena such as the change in tissue volume. To facilitate 3D imaging in this study, an incremental, quasi-static loading scheme used, which does not allow for exploration of the strain-rate dependence of the kinematic response. In addition, skin is multi-axially loaded *in vivo* and attached to the underlying fascia, conditions that undoubtedly put additional constraints on fiber kinematics not present in the uniaxial *in vitro* testing scheme. Finally, it is also important to note that these findings apply to mouse skin, which differs in many notable ways from human skin (Wei et al., 2017). Nonetheless, this study provides unique quantitative insights into skin kinematics at different scales that can not only aid in establishing more biofidelic computational models, but also provide a strong foundation to assess how changes in tissue structure can affect the mechanical properties of the tissue and clinical outcomes.

## Data Availability Statement

The raw data supporting the conclusions of this article will be made available by the authors, without undue reservation.

## Ethics Statement

The animal study was reviewed and approved by University of Arkansas IACUC Committee.

## Author Contributions

AW, ES, and KQ contributed to study conception and design. AW performed all data collection. JJ and NW helped with data analysis. JJ aided with animal work. AW drafted the manuscript. All authors contributed to manuscript revision and approved the final version.

## Conflict of Interest

The authors declare that the research was conducted in the absence of any commercial or financial relationships that could be construed as a potential conflict of interest.

## References

[B1] Alberto R. Draicchio F. Varrecchia T. Silvetti A. Iavicoli S. (2018). Wearable monitoring devices for biomechanical risk assessment at work: current status and future challenges-a systematic review. *Int. J. Environ. Res. Public Health* 15:2001. 10.3390/ijerph15092001 30217079 PMC6163390

[B2] Aubert L. Anthoine P. Rigal J. D. Leveque J. L. (1985). An in vivo assessment of the biomechanical properties of human skin modifications under the influence of cosmetic products. *Int. J. Cosmet. Sci.* 7 51–59. 10.1111/j.1467-2494.1985.tb00396.x 19460014

[B3] Ban E. Wang H. Franklin J. M. Liphardt J. T. Janmey P. A. Shenoy V. B. (2019). Strong triaxial coupling and anomalous Poisson effect in collagen networks. *Proc. Natl. Acad. Sci. U.S.A.* 116 6790–6799. 10.1073/pnas.1815659116 30894480 PMC6452734

[B4] Bancelin S. Aime C. Gusachenko I. Kowalczuk L. Latour G. Coradin T. (2014). Determination of collagen fibril size via absolute measurements of second-harmonic generation signals. *Nat. Commun.* 5:4920. 10.1038/ncomms5920 25223385

[B5] Bancelin S. Lynch B. Bonod-Bidaud C. Ducourthial G. Psilodimitrakopoulos S. Dokladal P. (2015). Ex vivo multiscale quantitation of skin biomechanics in wild-type and genetically-modified mice using multiphoton microscopy. *Sci. Rep.* 5:17635. 10.1038/srep17635 26631592 PMC4668561

[B6] Billiar K. L. Sacks M. S. (1997). A method to quantify the fiber kinematics of planar tissues under biaxial stretch. *J. Biomech.* 30 753–756. 10.1016/s0021-9290(97)00019-5 9239558

[B7] Blair M. J. Jones J. D. Woessner A. E. Quinn K. P. (2020). Skin structure-function relationships and the wound healing response to intrinsic aging. *Adv. Wound Care (New Rochelle)* 9 127–143. 10.1089/wound.2019.1021 31993254 PMC6985772

[B8] Campagnola P. J. Dong C. Y. (2011). Second harmonic generation microscopy: principles and applications to disease diagnosis. *Laser Photonics Rev.* 5 13–26. 10.1002/lpor.200910024

[B9] Campagnola P. J. Millard A. C. Terasaki M. Hoppe P. E. Malone C. J. Mohler W. A. (2002). Three-dimensional high-resolution second-harmonic generation imaging of endogenous structural proteins in biological tissues. *Biophys. J.* 82(1 Pt 1) 493–508. 10.1016/S0006-3495(02)75414-3 11751336 PMC1302489

[B10] Crichton M. L. Chen X. Huang H. Kendall M. A. (2013). Elastic modulus and viscoelastic properties of full thickness skin characterised at micro scales. *Biomaterials* 34 2087–2097. 10.1016/j.biomaterials.2012.11.035 23261214

[B11] Ducourthial G. Affagard J. S. Schmeltz M. Solinas X. Lopez-Poncelas M. Bonod-Bidaud C. (2019). Monitoring dynamic collagen reorganization during skin stretching with fast polarization-resolved second harmonic generation imaging. *J. Biophotonics* 12:e201800336. 10.1002/jbio.201800336 30604478

[B12] Elliott D. M. Narmoneva D. A. Setton L. A. (2002). Direct measurement of the Poisson’s ratio of human patella cartilage in tension. *J. Biomech. Eng.* 124 223–228. 10.1115/1.1449905 12002132

[B13] Fackler K. Klein L. Hiltner A. (1981). Polarizing light microscopy of intestine and its relationship to mechanical behaviour. *J. Microsc.* 124(Pt 3) 305–311. 10.1111/j.1365-2818.1981.tb02494.x 7328641

[B14] Fan R. Sacks M. S. (2014). Simulation of planar soft tissues using a structural constitutive model: finite element implementation and validation. *J. Biomech.* 47 2043–2054. 10.1016/j.jbiomech.2014.03.014 24746842 PMC4047197

[B15] Fang F. Lake S. P. (2016). Modelling approaches for evaluating multiscale tendon mechanics. *Interface Focus* 6:20150044. 10.1098/rsfs.2015.0044 26855747 PMC4686236

[B16] Fisher G. J. Varani J. Voorhees J. J. (2008). Looking older: fibroblast collapse and therapeutic implications. *Arch. Dermatol.* 144 666–672. 10.1001/archderm.144.5.666 18490597 PMC2887041

[B17] Fung Y. C. (1967). Elasticity of soft tissues in simple elongation. *Am. J. Physiol.* 213 1532–1544. 10.1152/ajplegacy.1967.213.6.1532 6075755

[B18] Giles J. M. Black A. E. Bischoff J. E. (2007). Anomalous rate dependence of the preconditioned response of soft tissue during load controlled deformation. *J. Biomech.* 40 777–785. 10.1016/j.jbiomech.2006.03.017 16730737

[B19] Harris C. Stephens M. (1988). “A combined corner and edge detector,” in *Proceedings of the Alvey Vision Conference 1988*, ed. Taylor J. (Manchester: Alvey Vision Club), 23.1–23.6.

[B20] Hepworth D. G. Steven-fountain A. Bruce D. M. Vincent J. F. (2001). Affine versus non-affine deformation in soft biological tissues, measured by the reorientation and stretching of collagen fibres through the thickness of compressed porcine skin. *J. Biomech.* 34 341–346. 10.1016/s0021-9290(00)00183-4 11182125

[B21] Hussain S. H. Limthongkul B. Humphreys T. R. (2013). The biomechanical properties of the skin. *Dermatol. Surg.* 39 193–203. 10.1111/dsu.12095 23350638

[B22] Jayyosi C. Affagard J. S. Ducourthial G. Bonod-Bidaud C. Lynch B. Bancelin S. (2017). Affine kinematics in planar fibrous connective tissues: an experimental investigation. *Biomech. Model. Mechanobiol.* 16 1459–1473. 10.1007/s10237-017-0899-1 28357604

[B23] Jor J. W. Parker M. D. Taberner A. J. Nash M. P. Nielsen P. M. (2013). Computational and experimental characterization of skin mechanics: identifying current challenges and future directions. *Wiley Interdiscip. Rev. Syst. Biol. Med.* 5 539–556. 10.1002/wsbm.1228 23757148

[B24] Lake S. P. Cortes D. H. Kadlowec J. A. Soslowsky L. J. Elliott D. M. (2012). Evaluation of affine fiber kinematics in human supraspinatus tendon using quantitative projection plot analysis. *Biomech. Model Mechanobiol.* 11 197–205. 10.1007/s10237-011-0303-5 21461899 PMC3644306

[B25] Lake S. P. Miller K. S. Elliott D. M. Soslowsky L. J. (2010). Tensile properties and fiber alignment of human supraspinatus tendon in the transverse direction demonstrate inhomogeneity, nonlinearity, and regional isotropy. *J. Biomech.* 43 727–732. 10.1016/j.jbiomech.2009.10.017 19900677 PMC2823853

[B26] Lanir Y. (1979). A structural theory for the homogeneous biaxial stress-strain relationships in flat collagenous tissues. *J. Biomech.* 12 423–436. 10.1016/0021-9290(79)90027-7 457696

[B27] Lee C. H. Zhang W. Liao J. Carruthers C. A. Sacks J. I. Sacks M. S. (2015). On the presence of affine fibril and fiber kinematics in the mitral valve anterior leaflet. *Biophys. J.* 108 2074–2087. 10.1016/j.bpj.2015.03.019 25902446 PMC4407258

[B28] Lees C. Vincent J. F. Hillerton J. E. (1991). Poisson’s ratio in skin. *Biomed. Mater. Eng.* 1 19–23. 1842507

[B29] Limbert G. (2017). Mathematical and computational modelling of skin biophysics: a review. *Proc. Math Phys. Eng. Sci.* 473:20170257. 10.1098/rspa.2017.0257 28804267 PMC5549575

[B30] Liu Z. Pouli D. Sood D. Sundarakrishnan A. Hui Mingalone C. K. Arendt L. M. (2017). Automated quantification of three-dimensional organization of fiber-like structures in biological tissues. *Biomaterials* 116 34–47. 10.1016/j.biomaterials.2016.11.041 27914265 PMC5210183

[B31] Lynch B. Bancelin S. Bonod-Bidaud C. Gueusquin J. B. Ruggiero F. Schanne-Klein M. C. (2017a). A novel microstructural interpretation for the biomechanics of mouse skin derived from multiscale characterization. *Acta Biomater.* 50 302–311. 10.1016/j.actbio.2016.12.051 28043893

[B32] Lynch B. Bonod-Bidaud C. Ducourthial G. Affagard J. S. Bancelin S. Psilodimitrakopoulos S. (2017b). How aging impacts skin biomechanics: a multiscale study in mice. *Sci. Rep.* 7:13750. 10.1038/s41598-017-13150-4 29061975 PMC5653787

[B33] Lynch H. A. Johannessen W. Wu J. P. Jawa A. Elliott D. M. (2003). Effect of fiber orientation and strain rate on the nonlinear uniaxial tensile material properties of tendon. *J. Biomech. Eng.* 125 726–731. 10.1115/1.1614819 14618932

[B34] Meador W. D. Sugerman G. P. Story H. M. Seifert A. W. Bersi M. R. Tepole A. B. (2020). The regional-dependent biaxial behavior of young and aged mouse skin: a detailed histomechanical characterization, residual strain analysis, and constitutive model. *Acta Biomater.* 101 403–413. 10.1016/j.actbio.2019.10.020 31614209

[B35] Nikishkov G. (2010). *Programming Finite Elements in Java^TM^.* London: Springer.

[B36] Oñate E. (2009). *Structural Analysis with the Finite Element Method.* Netherlands: Springer.

[B37] Pensalfini M. Haertel E. Hopf R. Wietecha M. Werner S. Mazza E. (2018). The mechanical fingerprint of murine excisional wounds. *Acta Biomater.* 65 226–236. 10.1016/j.actbio.2017.10.021 29031511

[B38] Pissarenko A. Meyers M. A. (2020). The materials science of skin: analysis, characterization, and modeling. *Prog. Mater. Sci.* 110:100634. 10.1016/j.pmatsci.2019.100634

[B39] Pissarenko A. Yang W. Quan H. Brown K. A. Williams A. Proud W. G. (2019). Tensile behavior and structural characterization of pig dermis. *Acta Biomater.* 86 77–95. 10.1016/j.actbio.2019.01.023 30660003

[B40] Quinn K. P. Georgakoudi I. (2013). Rapid quantification of pixel-wise fiber orientation data in micrographs. *J. Biomed. Opt.* 18:046003. 10.1117/1.JBO.18.4.046003 PMC3639785 23552635

[B41] Quinn K. P. Golberg A. Broelsch G. F. Khan S. Villiger M. Bouma B. (2015). An automated image processing method to quantify collagen fibre organization within cutaneous scar tissue. *Exp. Dermatol.* 24 78–80. 10.1111/exd.12553 25256009 PMC4289465

[B42] Quinn K. P. Sullivan K. E. Liu Z. Ballard Z. Siokatas C. Georgakoudi I. (2016). Optical metrics of the extracellular matrix predict compositional and mechanical changes after myocardial infarction. *Sci. Rep.* 6:35823. 10.1038/srep35823 27819334 PMC5098140

[B43] Quinn K. P. Winkelstein B. A. (2008). Altered collagen fiber kinematics define the onset of localized ligament damage during loading. *J. Appl. Physiol. (1985)* 105 1881–1888. 10.1152/japplphysiol.90792.2008 18845780

[B44] Quinn K. P. Winkelstein B. A. (2009). Vector correlation technique for pixel-wise detection of collagen fiber realignment during injurious tensile loading. *J. Biomed. Opt.* 14:054010. 10.1117/1.3227037 19895112

[B45] Quinn K. P. Winkelstein B. A. (2010). Full field strain measurements of collagenous tissue by tracking fiber alignment through vector correlation. *J. Biomech.* 43 2637–2640. 10.1016/j.jbiomech.2010.05.008 20494363

[B46] Quinn K. P. Winkelstein B. A. (2011). Preconditioning is correlated with altered collagen fiber alignment in ligament. *J. Biomech. Eng.* 133:064506. 10.1115/1.4004205 21744935

[B47] Reese S. P. Maas S. A. Weiss J. A. (2010). Micromechanical models of helical superstructures in ligament and tendon fibers predict large Poisson’s ratios. *J. Biomech.* 43 1394–1400. 10.1016/j.jbiomech.2010.01.004 20181336 PMC2881222

[B48] Reese S. P. Weiss J. A. (2013). Tendon fascicles exhibit a linear correlation between Poisson’s ratio and force during uniaxial stress relaxation. *J. Biomech. Eng.* 135:34501. 10.1115/1.4023134 PMC3705861 24231817

[B49] Ridge M. D. Wright V. (1966). The directional effects of skin. *J. Invest. Dermatol.* 46 341–346. 10.1038/jid.1966.54 5936039

[B50] Roeder B. A. Kokini K. Robinson J. P. Voytik-Harbin S. L. (2004). Local, three-dimensional strain measurements within largely deformed extracellular matrix constructs. *J. Biomech. Eng.* 126 699–708. 10.1115/1.1824127 15796328

[B51] Roeder B. A. Kokini K. Sturgis J. E. Robinson J. P. Voytik-Harbin S. L. (2002). Tensile mechanical properties of three-dimensional type I collagen extracellular matrices with varied microstructure. *J. Biomech. Eng.* 124 214–222. 10.1115/1.1449904 12002131

[B52] Roeder B. A. Kokini K. Voytik-Harbin S. L. (2009). Fibril microstructure affects strain transmission within collagen extracellular matrices. *J. Biomech. Eng.* 131:031004. 10.1115/1.3005331 19154063

[B53] Roth V. Mow V. C. (1980). The intrinsic tensile behavior of the matrix of bovine articular cartilage and its variation with age. *J. Bone Joint Surg. Am.* 62 1102–1117. 7430196

[B54] Sacks M. S. (2003). Incorporation of experimentally-derived fiber orientation into a structural constitutive model for planar collagenous tissues. *J. Biomech. Eng.* 125 280–287. 10.1115/1.1544508 12751291

[B55] Sacks M. S. Smith D. B. Hiester E. D. (1997). A small angle light scattering device for planar connective tissue microstructural analysis. *Ann. Biomed. Eng.* 25 678–689. 10.1007/Bf02684845 9236980

[B56] Sander E. A. Lynch K. A. Boyce S. T. (2014). Development of the mechanical properties of engineered skin substitutes after grafting to full-thickness wounds. *J. Biomech. Eng.* 136:051008. 10.1115/1.4026290 PMC4023834 24356985

[B57] Sander E. A. Stylianopoulos T. Tranquillo R. T. Barocas V. H. (2009). Image-based multiscale modeling predicts tissue-level and network-level fiber reorganization in stretched cell-compacted collagen gels. *Proc. Natl. Acad. Sci. U.S.A.* 106 17675–17680. 10.1073/pnas.0903716106 19805118 PMC2764876

[B58] Sen C. K. Gordillo G. M. Roy S. Kirsner R. Lambert L. Hunt T. K. (2009). Human skin wounds: a major and snowballing threat to public health and the economy. *Wound Repair Regen.* 17 763–771. 10.1111/j.1524-475X.2009.00543.x 19903300 PMC2810192

[B59] Tower T. T. Neidert M. R. Tranquillo R. T. (2002). Fiber alignment imaging during mechanical testing of soft tissues. *Ann. Biomed. Eng.* 30 1221–1233. 12540198 10.1114/1.1527047

[B60] Voytik-Harbin S. L. Roeder B. A. Sturgis J. E. Kokini K. Robinson J. P. (2003). Simultaneous mechanical loading and confocal reflection microscopy for three-dimensional microbiomechanical analysis of biomaterials and tissue constructs. *Microsc. Microanal.* 9 74–85. 10.1017/S1431927603030046 12597789

[B61] Wahlsten A. Pensalfini M. Stracuzzi A. Restivo G. Hopf R. Mazza E. (2019). On the compressibility and poroelasticity of human and murine skin. *Biomech. Model. Mechanobiol.* 18 1079–1093. 10.1007/s10237-019-01129-1 30806838

[B62] Wei J. C. J. Edwards G. A. Martin D. J. Huang H. Crichton M. L. Kendall M. A. F. (2017). Allometric scaling of skin thickness, elasticity, viscoelasticity to mass for micro-medical device translation: from mice, rats, rabbits, pigs to humans. *Sci. Rep.* 7:15885. 10.1038/s41598-017-15830-7 29162871 PMC5698453

[B63] Weiss J. A. Maker B. N. Govindjee S. (1996). Finite element implementation of incompressible, transversely isotropic hyperelasticity. *Comp. Methods Appl. Mech. Eng.* 135 107–128. 10.1016/0045-7825(96)01035-3

[B64] Woessner A. E. McGee J. D. Jones J. D. Quinn K. P. (2019). Characterizing differences in the collagen fiber organization of skin wounds using quantitative polarized light imaging. *Wound Repair Regen.* 27 711–714. 10.1111/wrr.12758 31418977 PMC7000211

[B65] Yasui T. Yonetsu M. Tanaka R. Tanaka Y. Fukushima S. Yamashita T. (2013). In vivo observation of age-related structural changes of dermal collagen in human facial skin using collagen-sensitive second harmonic generation microscope equipped with 1250-nm mode-locked Cr:Forsterite laser. *J. Biomed. Opt.* 18:31108. 10.1117/1.JBO.18.3.031108 23212157

